# Structural basis for high specificity of octopine binding in the plant pathogen Agrobacterium tumefaciens

**DOI:** 10.1038/s41598-017-18243-8

**Published:** 2017-12-21

**Authors:** Armelle Vigouroux, Abbas El Sahili, Julien Lang, Magali Aumont-Nicaise, Yves Dessaux, Denis Faure, Solange Moréra

**Affiliations:** 1 Institute for Integrative Biology of the Cell (I2BC), CNRS CEA Univ. Paris-Sud, Université Paris-Saclay, Avenue de la Terrasse, Gif-sur-Yvette, 91198 France; 2 Present Address: IPS2, INRA, 91 190 Gif-sur-Yvette, France; 3 Present Address: NTU Institute for Structural Biology, Nanyang Technological University, Experimental Medicine Building, Singapore, 636921 Singapore

## Abstract

*Agrobacterium* pathogens of octopine- and nopaline-types force host plants to produce either octopine or nopaline compounds, which they use as nutrients. Two *Agrobacterium* ABC-transporters and their cognate periplasmic binding proteins (PBPs) OccJ and NocT import octopine and nopaline/octopine, respectively. Here, we show that both octopine transport and degradation confer a selective advantage to octopine-type *A*. *tumefaciens* when it colonizes plants. We report the X-ray structures of the unliganded PBP OccJ and its complex with octopine as well as a structural comparison with NocT and the related PBP LAO from *Salmonella enterica*, which binds amino acids (lysine, arginine and ornithine). We investigated the specificity of OccJ, NocT and LAO using several ligands such as amino acids, octopine, nopaline and octopine analogues. OccJ displays a high selectivity and nanomolar range affinity for octopine. Altogether, the structural and affinity data allowed to define an octopine binding signature in PBPs and to construct a OccJ mutant impaired in octopine binding, a selective octopine-binding NocT and a non-selective octopine-binding LAO by changing one single residue in these PBPs. We proposed the PBP OccJ as a major trait in the ecological specialization of octopine-type *Agrobacterium* pathogens when they colonize and exploit the plant host.

## Introduction

Opines are low molecular weight (200 to 600 g/mol) carbon compounds, characteristic of crown-gall tumors induced by pathogenic, soil-inhabiting *Agrobacterium* species. These include *A*. *tumefaciens*, *A*. *rhizogenes*, and *A*. *vitis*. Upon infection, agrobacteria transfer the so-called T-DNA from the tumor-inducing plasmid (pTi) into the host plant cells^1^. Once integrated into the nuclear genome of the host plant, T-DNA genes drive ecological niche-construction by inducing plant cell division to form a tumor within which opines are synthesized. Only *A*. *tumefaciens* bacteria that contain a Ti plasmid can catabolize opines, which are thus key players in *Agrobacterium* niche construction and exploitation^2,3^. Indeed, bacterial cells harboring a pTi can catabolize opines and outcompete cells with mutated Ti-plasmids lacking opine catabolic activity, formally demonstrating the validity of the opine concept^4–6^. Different types of pTi are known, they code for the synthesis and catabolism of opines with different molecular structures. Over twenty different opines have been described so far^7^. Amongst these, nopaline and octopine were the most extensively studied opines. Octopine synthase (Ocs) and nopaline synthase (Nos) are encoded by eponymous T-DNA genes, and responsible for the synthesis of compounds from the octopine and nopaline families in octopine and nopaline tumors, respectively^8–10^. They catalyse the reductive condensation of arginine with pyruvate (octopine) or α-ketoglutarate (nopaline). In contrast to nopaline synthase that can only condense arginine and ornithine with α-ketoglutarate (to form nopaline and nopalinic acid, respectively), octopine synthase can also condense other amino acids such as ornithine, lysine or histidine with pyruvate to form octopinic acid, lysopine and histopine, respectively. This latter set of molecules with other compounds, including sulfonopine^11^, defines the octopine family.

Recognition and import of opines are conferred by periplasmic binding proteins (PBPs) and their associated ATP-binding cassette (ABC) transporters. The PBP OccJ is associated with the octopine transporter of octopine-type *A*. *tumefaciens* while NocT is associated with nopaline uptake in the nopaline-type *A*. *tumefaciens*. Nonetheless, NocT can recognize both nopaline and octopine whereas OccJ accepts octopine but not nopaline^6,12,13^. Interestingly, nopaline-type strains are able to assimilate octopine, once nopaline induces the nopaline assimilative pathway^6^. We have recently shown that both octopine and nopaline can bind NocT with similar affinity in the micromolar range and we have characterized their binding mode to NocT at atomic level^14^. OccJ shares 47% sequence identity with NocT and belongs to cluster F within the PBP structural classification^15^. This cluster contains amino acid binding proteins including the well-described PBP LAO (Lysine-Arginine-Ornithine;^16,17^) from *Salmonella enterica*.

Combining microbiology, affinity measurements, structural biology and site directed mutagenesis, and using opines and analogues, we investigated the ligand binding specificity of three related PBPs OccJ, NocT and LAO. We identified critical residues involved in ligand interactions and further proposed an octopine binding signature. We used homooctopine, noroctopine, noroctopinic acid and homo-noroctopine as analogues of octopine family known to isolate regulatory mutants for the pTi-borne octopine utilisation genes^18,19^. Although the mutation of a single amino acid in NocT and LAO can modify their ligand selectivity to transform them into octopine selective and non-selective PBPs, respectively, our work shows that OccJ is evolved for binding octopine and octopinic acid with high affinity in nanomolar range mainly due to the presence of a serine at position 91. This very efficient binding ability may have contributed to the specialization of the octopine-type *A*. *tumefaciens*, and explains why the nopaline-type agrobacteria (generalists) which display a drastically lower affinity (micromolar range) for both nopaline and octopine do not spread despite the advantage associated with broader nutritional niches.

## Results

### The PBP OccJ is involved in octopine uptake *in vitro* and *in planta*

**Figure 1:**
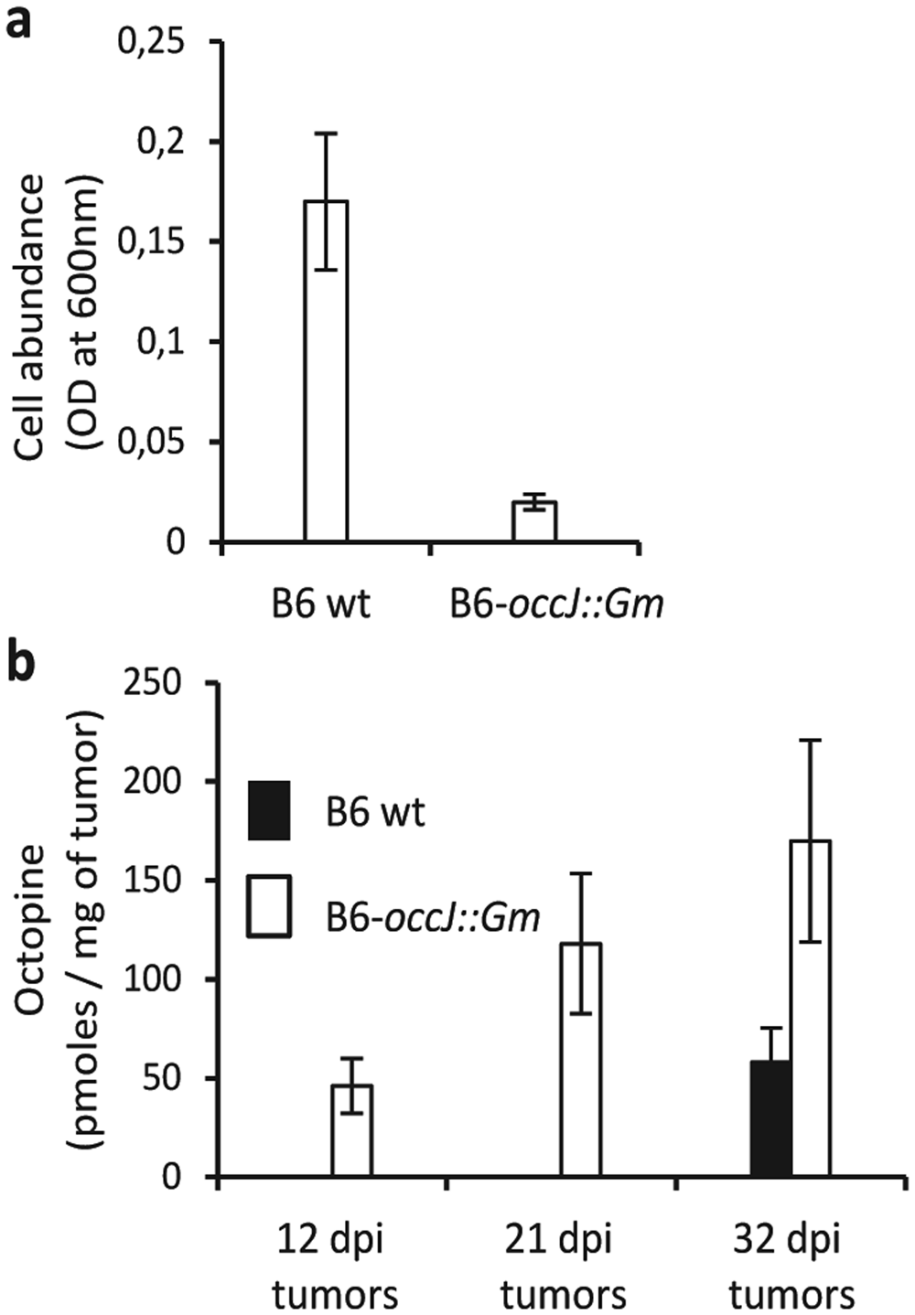
OccJ involvement in octopine consumption *in vitro* and in planta. (**a**) Overnight (16 hours) growth (OD at 600 nm) of *A*. *tumefaciens* WT B6 strain and B6-occJ::Gm mutant in AB minimal medium supplemented with octopine as a sole nitrogen and carbon source. (**b**) Abundance of octopine in tomato plant tumors (pmoles/mg of fresh weight plant tissues) induced by either *A*. *tumefaciens* WT B6 or B6-occJ::Gm mutant. Sampling was performed 12, 21 and 32 days post infection (dpi). In *in vitro* and in planta assays, mean values were calculated with four measurements collected from two independent experiments.

The growth profiles of the wild-type (WT) *A*. *tumefaciens* strain B6 and that of its derivative, B6-occJ::Gm, were compared in minimal medium containing octopine as the sole source of carbon and nitrogen. Under these conditions, proliferation of the mutant strain B6-occJ was drastically limited (Fig. 1a), indicating the involvement of OccJ and proteins encoded by downstream genes in octopine uptake and assimilation in pure culture. In plant tumors infected with either *A*. *tumefaciens* strain B6 WT or its derivative B6-occJ::Gm, the abundance of octopine was quantified. Octopine accumulated at a higher level in plant tumors induced by *A*. *tumefaciens* B6-occJ::Gm as compared to those induced by *A*. *tumefaciens* B6 WT (Fig. 1b), implying an impaired exploitation of the octopine resource in planta by the OccJ-defective mutant.

### The PBP OccJ conferred a competitive advantage in colonizing plant tumor

**Figure 2:**
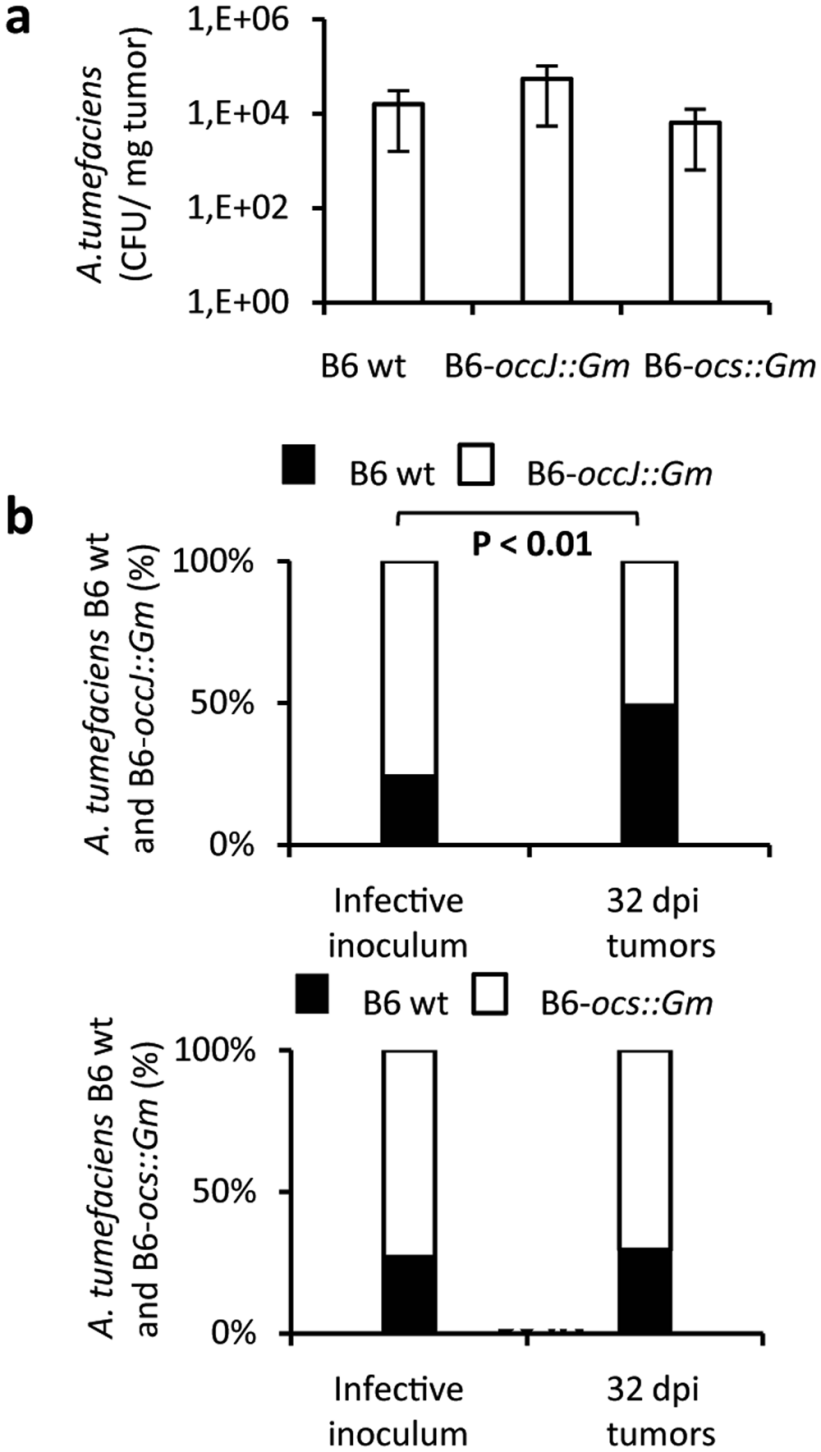
OccJ confers a competitive advantage in plant tumor. (**a**) *A*. *tumefaciens* bacterial concentration (CFU/mg fresh weight tumor) in tomato tumor (at 32 dpi) infected with either *A*. *tumefaciens* WT B6 or B6-occJ::Gm or B6-ocs::Gm. (**b**) Proportion of *A*. *tumefaciens* genotypes (%) in inoculum and tomato tumor (at 32 dpi) infected with a mixture of *A*. *tumefaciens* WT B6 and B6-occJ::Gm (top graph) or *A*. *tumefaciens* WT B6 and B6-ocs::Gm (bottom graph). Fisher’s exact test was used for the comparison of genotype relative abundance in inoculum versus plant tumor. Values were collected from two independent experiments, each conditions involving from 5 to 7 plant tumors.

The colonization of plant tumor by *A*. *tumefaciens* B6 WT and B6-occJ::Gm was evaluated. In this experiment, we also used another *A*. *tumefaciens* B6 mutant, B6-ocs::Gm, harboring a disrupted version of the octopine synthase gene (*ocs*) in the T-DNA transferred to tumor plant cells. While the mutant B6-ocs::Gm is impaired in octopine-niche construction, the mutant B6-occJ::Gm is impaired in octopine-niche exploitation. When tomato plants were infected with *A*. *tumefaciens* B6 WT, B6-occJ::Gm or B6-ocs::Gm individually, the bacterial concentration in tumors induced by each strain did not differ (around 10^4^ UFC/mg of tumor), showing that each genotype was able to colonize the plant tumor irrespective of the construction or exploitation of the octopine niche (Fig. 2a). This could be explained by the presence of other nutrients in the plant tumors. By contrast, when *A*. *tumefaciens* B6 (WT) and B6-occJ::Gm were co-inoculated in a 20:80 (WT:mutant) inoculum ratio in a same tumor, a reduced fitness was observed for B6-occJ::Gm (Fig. 2b), revealing a selective advantage conferred by octopine-niche exploitation under a competitive challenge. In contrast, when *A*. *tumefaciens* B6 WT and B6-ocs::Gm were co-inoculated, no variation of the relative abundance of the two genotypes was observed, each being able to exploit the octopine-niche constructed by *A*. *tumefaciens* B6 WT.

### OccJ exhibits a very high affinity for octopine in a nanomolar range

Ligand binding to the protein OccJ was investigated using tryptophan fluorescence spectroscopy, a method exploiting significant environmental changes accompanying the binding, and/or isothermal titration microcalorimetry (ITC). Titration experiments yielded an apparent dissociation constant *K*_*D*_ value of 4 ± 1 nM for octopine, similar to that obtained by ITC (Table 1 and Supplementary Fig. S1). ITC confirmed the 1:1 binding stoichiometry. NocT also binds octopine, but displays a 1,000 fold lower affinity with a *K*_*D*_ of 6.1 ± 0.7 µM in the same range as for nopaline (*K*_*D*_ of 3.7 ± 0.6 µM;^6,14^). Because LAO’s two tryptophan residues lie far from the ligand binding site, no fluorescence intensity change was detected. We therefore relied upon ITC to measure ligand affinity. No interaction between LAO and octopine could be detected while a high affinity for arginine was determined with a *K*_*D*_ value of 24 ± 2 nM (Table 1 and Supplementary Fig. S1) in line with the previous reported affinity for arginine, lysine and ornithine at a nanomolar range^20^. Whereas NocT does not interact with arginine, lysine or ornithine^6^, OccJ binds these three amino acids with micromolar affinity with a preference for the longest side chains (Table 1 and Supplementary Fig. S1).

**Table 1:** Opines and amino acids affinity measurement for OccJ, NocT, LAO and mutants. *K*_*D*_ values were measured by intrinsic protein fluorescence titration (F) and/or by isothermal titration microcalorimetry (ITC). NI means no interaction whereas a white case means experiment not done.

Protein		Octopine *K*_*D*_ μM	Nopaline *K*_*D*_ μM	L-arginine *K*_*D*_ μM	L-lysine *K*_*D*_ μM	ornithine *K*_*D*_ μM
OccJ WT	F/ITC	0.004 ± 0.0001/0.009	NI	7.2 ± 1/35.7 ± 2.1	7.5 ± 2.1/71 ± 6	31.2 ± 3.5/40.5 ± 15
OccJ S91G	F/ITC	29.9 ± 3.8/20 ± 3	NI	NI		
OccJ N202D	F/ITC	0.006 ± 0.001/0.016 ± 0.008	NI	7.5 ± 1.2/20.4 ± 4		
NocT WT	F/ITC	6.1 ± 0.7/9.9 ± 0.7	3.7 ± 0.6/	NI	NI	NI
NocT G97S	F/ITC	63.8 ± 13.8/52 ± 10	NI	NI		
LAO WT	ITC	NI	NI	0.024 ± 0.002		
LAO Q122A	ITC	34 ± 5	NI	0.12 ± 0.01		

### OccJ fold is a PBP from cluster F

**Figure 3:**
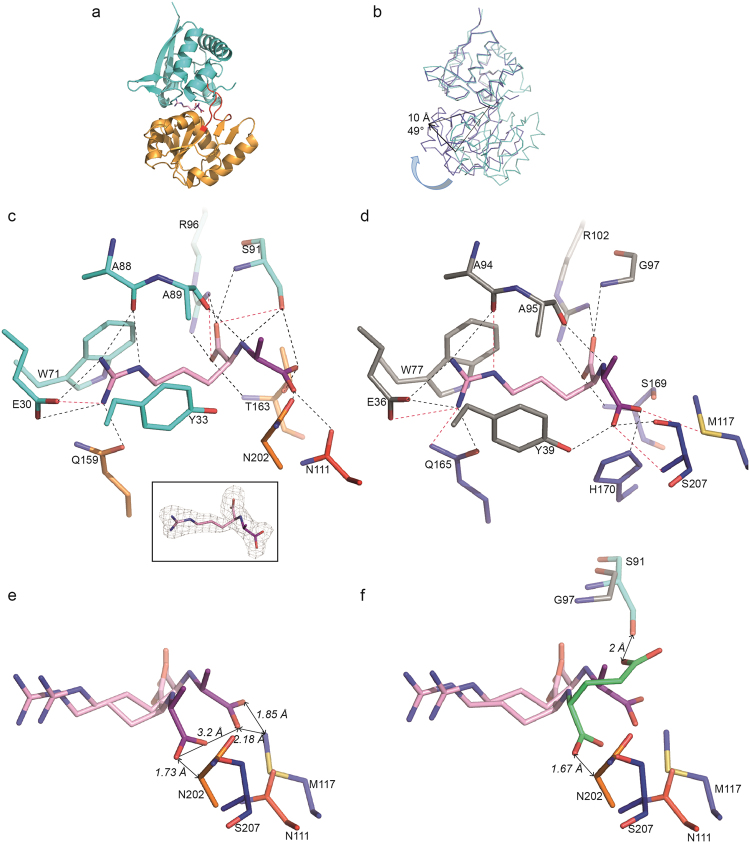
Ribbon representation of OccJ structures with octopine in pink/magenta for the arginine/pyruvate part, respectively. (**a**) Lobes 1 and 2 are shown in cyan and orange, respectively, and the hinge region in red. (**b**) Comparison between the open unliganded (cyan) and the closed liganded (deep blue) forms of OccJ. A curved arrow indicates the movement of lobe 2 upon ligand binding (**c**) octopine bound to the ligand binding site of OccJ in its annealing Fo-Fc omit map contoured at 4 σ. Hydrogen bonds between octopine and OccJ are shown as dashed lines in black (distances below 3.2 Å) and in magenta (distances between 3.2 and 3.4 Å) (**d**) octopine bound to the ligand binding site in NocT-octopine structure^14^ (**e**) closed-up view around the pyruvate moiety of octopine from the superposition of OccJ-octopine and NocT-octopine complexes. Double arrows indicate distances. (**f**) Closed-up view around the pyruvate moiety and the α-ketoglutarate of octopine and nopaline shown in pink/green, respectively, from the superposition of OccJ-octopine and NocT-nopaline complexes. Double arrows indicate distances.

The mature PBP OccJ (256 aa without the signal peptide) expression plasmid was obtained by cloning the *occJ* gene lacking the first twenty residues of the signal sequence that serves for localization to bacterial periplasm. The X-ray structures of the unliganded and liganded OccJ with octopine were solved at 2.35 and 2 Å resolution respectively (Table 2). The liganded crystal contained two very similar molecules in the asymmetric unit as indicated by the overall root mean square deviations (RMSD) for all Cα atoms of 0.28 Å. The liganded form adopts a closed conformation (Fig. 3a) whereas the unliganded form adopts an open conformation. Indeed, superposition of the two structures results in an average RMSD of 4 Å for all Cα atoms and a structural comparison shows a 49° rotation around the hinge region of the C-terminal domain (residues 112–226) upon ligand binding once the N-terminal domains (residues 21–107 and 234–276) were superimposed leading to a movement of 10 Å for Thr163 (Fig. 3b). As expected, the most similar overall structures (SSM-EBI: http://www.ebi.ac.uk/msd-srv/ssm) are PBPs from the same cluster F with the best hit for the liganded structures of NocT (average RMSD of 1.6 Å over all Cα atoms) used as a search model for the molecular replacement. Similarly to NocT, OccJ shares 35% sequence identity with LAO and a RMSD value of 1.8 Å for 228 Cα atoms.

**Table 2:** Crystallographic data and refinement parameters.

PDB code	OccJ	OccJ-octopine	NocT-G97S- octopine	NocT-octopinic acid	NocT-noroctopinic acid	NocT-histopine
	5ORE	5ORG	5OT8	5OTA	5OTC	5OT9
Precipitant	20% P4K/ 0.1 M Tris pH 8.5/0.2 M acetate NH_4_	25% P4K/0.1 M Tris pH 8.5/0.1 M acetate NH_4_	30% P4K/0.1 M Tris pH 8/0.1 M LiSO_4_	30% P4K/0.1 M Tris pH 8/0.1 M LiSO_4_	30% P4K/0.1 M Tris pH 8/0.1 M LiSO_4_	30% P4K/0.1 M Tris pH 8/0.1 M LiSO_4_
Space group; Cell parameters (Å,°)	*P4*_3_2_1_ *2; a* = 59.3 *b* = 59.3 *c* = 125.99	*P4*_3_2_1_2; *a* = 99.5 *b* = 99.5 *c* = 157.6	*P3*_2_; *a* = 115.7 *b* = 115.7 *c* = 38.0	*P3*_2_; *a* = 115.2 *b* = 115.2 *c* = 37.8	*P3*_2_; *a* = 114 *b* = 114 *c* = 37.8	*P3*_*2*_; *a* = 113.8 *b* = 113.8 *c* = 37.8
Resolution (Å)	50–2.35 (2.49–2.35)	30–2 (2.4–2)	50–2.35 (2.49–2.35)	50–2.1 (2.22–2.1)	50–2.2 (2.33–2.2)	50–2.45 (2.59–2.45)
No. of observed reflections	271394 (42359)	354632 (43838)	107030 (17196)	188563 (29380)	129530 (20180)	82163 (12932)
No. of unique reflections	9977 (1558)	54705 (8556)	23735 (3814)	32842 (5234)	27945 (4445)	20178 (3227)
R_*sym*_ (%)	14 (212.6)	8.8 (101.9)	9.3 (71.5)	9.6 (86.2)	7.5 (119.6)	8.8 (84.7)
Completeness (%)	99.9 (99.3)	99.6 (98.3)	99.8 (99.7)	99.7 (98.4)	99.5 (97.5)	99.7 (98.9)
I/σ	20.8 (1.7)	14 (1.7)	11.6 (1.8)	13.2 (1.8)	14.1 (1.3)	10.3 (1.6)
CC _1/2_	99.9 (67.3)	99.8 (62.3)	99.7 (71.5)	99.8 (65.5)	99.9 (50.8)	99.6 (57.1)
R_*cryst*_ (%)	20.8	19.5	18.3	17.7	19	19.2
R_*free*_ (%)	25.1	22	20.5	20.4	20.7	22.3
rms bond deviation (Å)	0.01	0.01	0.009	0.01	0.01	0.009
rms angle deviation (°)	1.26	1.12	1.08	1.08	1.06	1.07
Average B (Å^2^) protein/ligand/solvent	68.7/ /73.3	46/31.9/51.9	51.7/48.5/57.4	40.6/30.3/ 45	60.1/60.1/66.2	69.8/61/67.2

Values in parenthesis are those for the last shell. CC_1/2_ = percentage of correlation between intensities from random half‐dataset (P. A. Karplus, K. Diederichs, Science 2012, 336, 1030–1033).

### Structural comparison between OccJ-octopine and NocT-octopine complexes: a different octopine binding mode

The octopine bound between the two closed lobes of OccJ is very well defined in the electron density maps (Fig. 3c), and is surrounded by 18 residues defining the ligand binding site of OccJ (Table 3). Both structures of OccJ and NocT in complex with octopine (PDB code 5ITP for NocT-octopine,^14^) superimpose with an average RMSD of 1.7 Å over all Cα atoms. They share a very similar binding site around the arginine moiety of octopine (Table 3 and Fig. 3c,d). Indeed, the guanidyl side chain of arginine is wedged between two conserved aromatic residues (Tyr33/39 and Trp71/77 in OccJ/NocT) and points toward the opening of the cleft by making six hydrogen bonds with the conserved side chains of residues Glu30/36 and Gln159/165 and the carbonyl of Ala88/94. Its carboxyl moiety makes a salt-bridge with the conserved Arg96/102 and three hydrogen bonds with the Ser91 side chain (the corresponding residue in NocT is Gly97) and the amide NH protons of Ser91/Gly97 and Thr163/Ser169. Its amide NH proton interacts with the carbonyl of Ala89/95 and the side chain of Ser91 in OccJ. Therefore, there is a unique difference on the arginine moiety binding between OccJ and NocT: Ser91 versus Gly97, respectively.

**Table 3:** Comparison of residues at the binding site of *A*. *tumefaciens* (At) OccJ and NocT and *S*. *enteratica* (Se) LAO. Residues in bold show those that are different between OccJ, NocT and LAO. * are residues interacting with the ligand through hydrophobic stacking, van der Waals contacts and/or hydrogen bonding. Residues in *italics * and underlined are the binding signature for the pyruvate moiety of octopine in OccJ and the ketoacid moiety of octopine/nopaline in NocT, respectively.

OccJ (At)	NocT (At)	LAO (Se)
Glu30*	Glu36*	Asp11*
Tyr33*	Tyr39*	Tyr14*
Trp36	Tyr42	Phe17
Trp71*	Trp77*	Phe52*
Ala88*	Ala94*	Ser69*
Ala89*	Ala95*	Ser70*
Ser91*	Gly97*	Ser72*
Arg96*	Arg102*	Arg77*
Gly109	Thr115	Ala90
***Asn111*** *	**Met117** *	**Ser92**
Gln159*	Gln165*	Leu117*
Thr162	Thr168	Ser120
Thr163*	Ser169*	Thr121*
***Ala164***	His170*	**Gln122**
Asn202*	Ser207*	Asp161*
Phe230	Phe235	Phe191

In contrast, there are many differences for the binding of pyruvate moieties of octopine between OccJ and NocT, affecting their conformation and their protein interactions. Indeed, they do not overlap and are 3.2 Å apart when superimposed (Fig. 3e). In OccJ, the pyruvate moiety interacts with four non-conserved side chains: Ser91, Asn111, Thr163 and Asn202, which correspond to Gly97, Met117, Ser169 and Ser207 in NocT. Met117 and Ser207 in NocT interact with the pyruvate carboxylate in contrast to Ser169 that is too far from it. Ser91 in OccJ once again appears as a major difference in the binding site of the pyruvate moiety of octopine in OccJ compared with NocT, making this residue potentially critical for octopine binding. Indeed, Ser91 in OccJ plays a remarkable role in holding octopine in three parts of the molecule, including both carboxyl groups. Ser91 is around 2 Å from the α-ketoglutarate of nopaline upon superposition with the 1.75 Å resolution NocT-nopaline structure that we solved in this study (Table S1; Fig. 3f). This structure is very similar to that published at 2.3 Å resolution (PDB code 4POX, RMSD of 0.2 Å for all Cα atoms). As previously mentioned in Lang *et al*.^14^, Ser91 seems incompatible with a possible binding of nopaline to OccJ due to steric hindrance. The side chain of Asn202 in OccJ also does not seem in a suitable position to accommodate nopaline or octopine in the conformation observed in NocT because its C_β_ atom is less than 2 Å from nopaline (Fig. 3e,f).

### Ser91 in OccJ is a key residue for an efficient octopine binding and a corresponding serine in NocT transforms NocT to a selective octopine binding protein

To validate the key role of Ser91 in OccJ, we produced the OccJ-S91G mutant in which Ser91 was replaced by a glycine residue corresponding to Gly97 in NocT. Using intrinsic protein fluorescence titration, OccJ-S91G still binds octopine but with a drastically (over 7,000-fold) lower affinity than that of OccJ (Table 1 and Supplementary Fig. S1). In contrast to the WT OccJ, this mutant can no longer bind arginine, lysine or ornithine, and nopaline is not a ligand of this mutant as for the WT.

In parallel, we produced the corresponding reciprocal mutant in NocT, in which Gly97 was replaced by a serine. As expected, the presence of the serine at position 97 in NocT abolished the nopaline binding ability whereas octopine binding was preserved but with a 10-fold lower affinity compared with the WT (Table 1 and Supplementary Fig. S1). Therefore, in contrast to OccJ, a serine at position 97 does not favour high affinity for octopine in NocT. We thus validated that the presence of Gly97 in NocT was essential for the spatial accommodation of the α-ketoglutarate moiety of nopaline.

We solved the structure of NocT-G97S in complex with octopine at 2.35 Å. Both structures of the WT NocT (PDB code 5ITP) and NocT-G97S mutant in complex with octopine are similar with an average RMSD of 0.25 Å for all Cα atoms. Remarkably, both octopine molecules overlap and adopt a similar conformation, unlike observations in OccJ (Supplementary Fig. S2a). Both structures, together with that previously reported of NocT-M117N in complex with octopine, (PDB code 5ITO,^14^) clearly show that each bound octopine adopts a similar conformation, making similar protein interactions with residues at positions 117, 170 and 207. Therefore, the G97S mutation has a low impact on the protein mutant-octopine interactions compared with the WT, in line with affinity towards octopine in the same range (Supplementary Fig. S2b). Moreover, Ser97 exhibits different conformations in both molecules of the asymmetric unit of the NocT-G97S-octopine structure and each of it is not compatible with a bound nopaline (Supplementary Fig. S2c).

### A single mutation in LAO can transform LAO as an octopine binding protein

**Figure 4:**
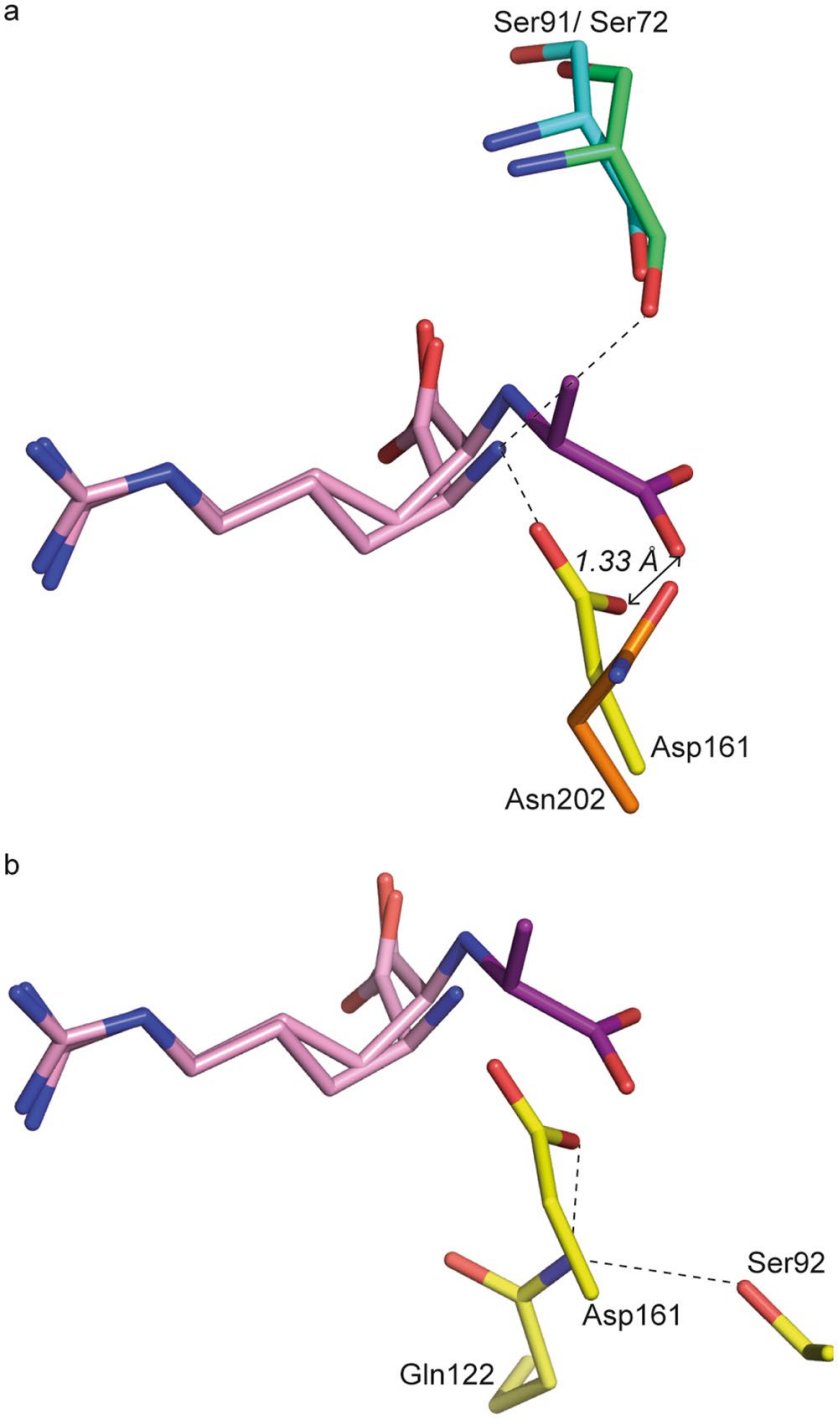
Superposition of OccJ and LAO binding site showing octopine in pink/magenta in OccJ and arginine in pink in LAO (PDB code 1LAF). (**a**) Ser72 and Asp161 in LAO (Ser91 and Asn202 in OccJ) bind the NH_3_^+^ group of the arginine ligand. Asp161 side chain position is not compatible with a bound octopine in LAO. Double arrows indicate distances. (**b**) Asp161 side chain in LAO is hydrogen bonded with Gln122, which in turn interacts with Ser92.

OccJ and LAO bind similarly the side chain of the arginine ligand and its carboxyl group (Table 3). Nonetheless, the amino group of the arginine ligand is tightly bound in LOA with the side chains of Ser72 (corresponding to Ser91 in OccJ) and Asp161 (Asn202 in OccJ). Importantly, the major ionic interaction is missing in OccJ leading to less efficient binding of the arginine ligand, in line with the micromolar affinity versus nanomolar affinity for LAO. The presence of Asp161 in LAO (Asn202 in OccJ) seems incompatible with an octopine binding ability, due to steric clash upon superposition of OccJ-octopine complex and LAO-arginine complex (PDB code 1LAF, Fig. 4a). In agreement, LAO cannot bind octopine. To validate this hypothesis, we produced the OccJ-N202D mutant in which Asn202 was replaced by an aspartate residue. Affinity measurements by different techniques suggest that OccJ-N202D is similar to the WT with respect to binding of octopine and arginine, and demonstrate that the presence of this aspartate in OccJ has no effect on the ligand recognition (Table 1 and Supplementary Fig. S1). An aspartate at position 202 in the OccJ-octopine complex was modelled and its carboxylate group is free and positioned far from the pyruvate to be compatible with a bound octopine. In contrast, the Asp161 side chain in LAO is tightly bound by a hydrogen bond with Gln122 side chain (Ala164 in OccJ) which in turn interacts with Ser92 side chain (Asn111 in OccJ) (Fig. 4b). In LAO, Asp161, Gln122 and Ser92 seem to form a rigid template in the ligand binding site. Removing Gln122 in the LAO-Q122A mutant (corresponding to Ala164 in OccJ) results in a lower affinity of 5-fold toward arginine compared with WT and allows the binding of octopine with a K_*D*_ of 34 ± 5 μM underlining the need to release the Asp161 side chain in LAO to accommodate octopine (Table 1 and Supplementary Fig. S1).

### Binding of natural octopine family members and octopine analogues to OccJ and NocT

Using autofluorescence, OccJ displays K_*D*_ values of 17 ± 3 nM and 140 ± 10 nM for octopinic acid and lysopine, respectively. In contrast, the binding affinity for histopine was drastically weaker, in the micromolar range (Table 4 and Supplementary Fig. S3). No fluorescence intensity change was detected by incubating OccJ with histidine or nopaline. OccJ also exhibited a 10-fold better affinity for noroctopine and noroctopinic acid compared with arginine or ornithine alone (Tables 1 and 4), supporting the idea that the addition of glyoxylic acid (one CH_3_ shorter than pyruvate) considerably enhances the binding affinity of the opine analogues. Replacing the arginine in the noroctopinic acid by a homoarginine (a longer residue than arginine due to an additional CH_2_ in the side chain) resulted in a lower affinity, in the micromolar range. Using ITC, we confirmed the affinity results obtained by autofluorescence (Table 4 and Supplementary Fig. S3) and the 1:1 binding stoichiometry. The negative enthalpy change upon each ligand binding suggests that the binding is enthalpy driven, and similar binding isotherms for all ligands imply the same binding mechanism involving polar interactions. The same study performed with NocT showed that NocT displays similar affinity for octopine and its family members except for histopine, which is the worst ligand as for OccJ. The homooctopine analogue behaves like octopine. NocT binds the three other octopine analogues with a slightly lower affinity, in the tens of micromolar.

**Table 4:** Affinity measurement for OccJ and NocT towards natural octopine family members and octopine analogues. ITC experiments of NocT were not performed with lysopine and histopine.

	Ligand	F	ITC					
		Kd (µM)	Kd (µM)	N	Enthalpy (ΔH) (cal/mol)	Entropy (ΔS) (cal/mol/deg)	Entropic contribution (-TΔS) (cal/mol)	Free enthalpy (ΔG) (cal/mol)
OccJ	octopinic acid	0.017 ± 0.003	0.038 ± 0.007	0.87	−9547	1.40	−410	−9957
	lysopine	0.14 ± 0.01	0.11 ± 0.02	0.97	−4832	15.2	−4455	−9287
	histopine	33.1 ± 3.5	326	1.03	−854	13	−3810	−4664
	homo-octopine	0.23 ± 0.03	0.53 ± 0.04	0.93	−7611	2.75	−806	−8417
	noroctopine	0.19 ± 0.05	0.15 ± 0.01	1.02	−4804	14.8	−4367	−9171
	noroctopinic acid	0.23 ± 0.4	0.15 ± 0.01	0.92	−7202	6.60	−1934	−9136
	homo-noroctopine	4.63 ± 0.42	19.3 ± 1.4	1.01	−4115	7.53	−2207	−6322
NocT	octopinic acid	1.8 ± 0.3	4.4 ± 0.4	0.87	−3658	12.0	−3517	−7175
	lysopine	11.2 ± 3.5						
	histopine	84.4 ± 6.7						
	homo-octopine	5.4 ± 0.6	30.6 ± 2.1	0.86	−3832	7.58	−2222	−6054
	noroctopine	33.4 ± 2.7	78 ± 9	0.9	872	21.8	−6390	−5518
	noroctipinic acid	19.3 ± 1.37	23 ± 5	0.92	620	23.3	−6830	−6210
	homo-noroctopine	21.3 ± 2.1	79 ± 14	0.75	−1195	14.7	−4309	−5504

OccJ needs to be highly concentrated for co-crystallization assays (above 100 mg/mL) as the addition of octopine to OccJ leads to a high solubility of the complex. Thus, it has been very difficult to obtain OccJ crystals in complex with octopine because a massive amount of purified protein was required for this experiment. No crystals appeared with other opines or analogues. In contrast, we were successful in solving the structures of NocT with octopinic acid, noroctopinic acid and histopine at 2.1, 2.2 and 2.45 Å resolution (Table 2), respectively. This allowed us to validate the structure and binding mode of each compound to NocT. All ligands are well defined in their electron density maps (Supplementary Fig. S4). Octopinic acid and noroctopinic acid contain an ornithine moiety instead of the arginine for octopine. Local rearrangements of residues in the ligand binding site such as Glu36, Ala94, Met117 and those from the region 165–170 occur upon octopinic acid, noroctopinic acid and histopine binding compared with octopine, with Glu36 and Gln165 moving towards both shortest side chains of ornithine and histidine moieties. The ornithine moiety forms fewer polar interactions with the protein than the arginine moiety. This is also the case for histidine in histopine. Moreover both hydrogen bonds between histidine moiety and NocT are around 3–3.1 Å, likely explaining the weak affinity of NocT for histopine compared to the other compounds having arginine and ornithine (Supplementary Fig. S4a–c). In contrast, the pyruvate of the octopinic acid and histopine as well as the glyoxylic acid (a CH_3_ shorter than pyruvate) of noroctopinic acid adopt a similar position, comparable to that of the pyruvate of octopine (Supplementary Figs. S4d).

### OccJ is present in a few bacteria

**Figure 5:**
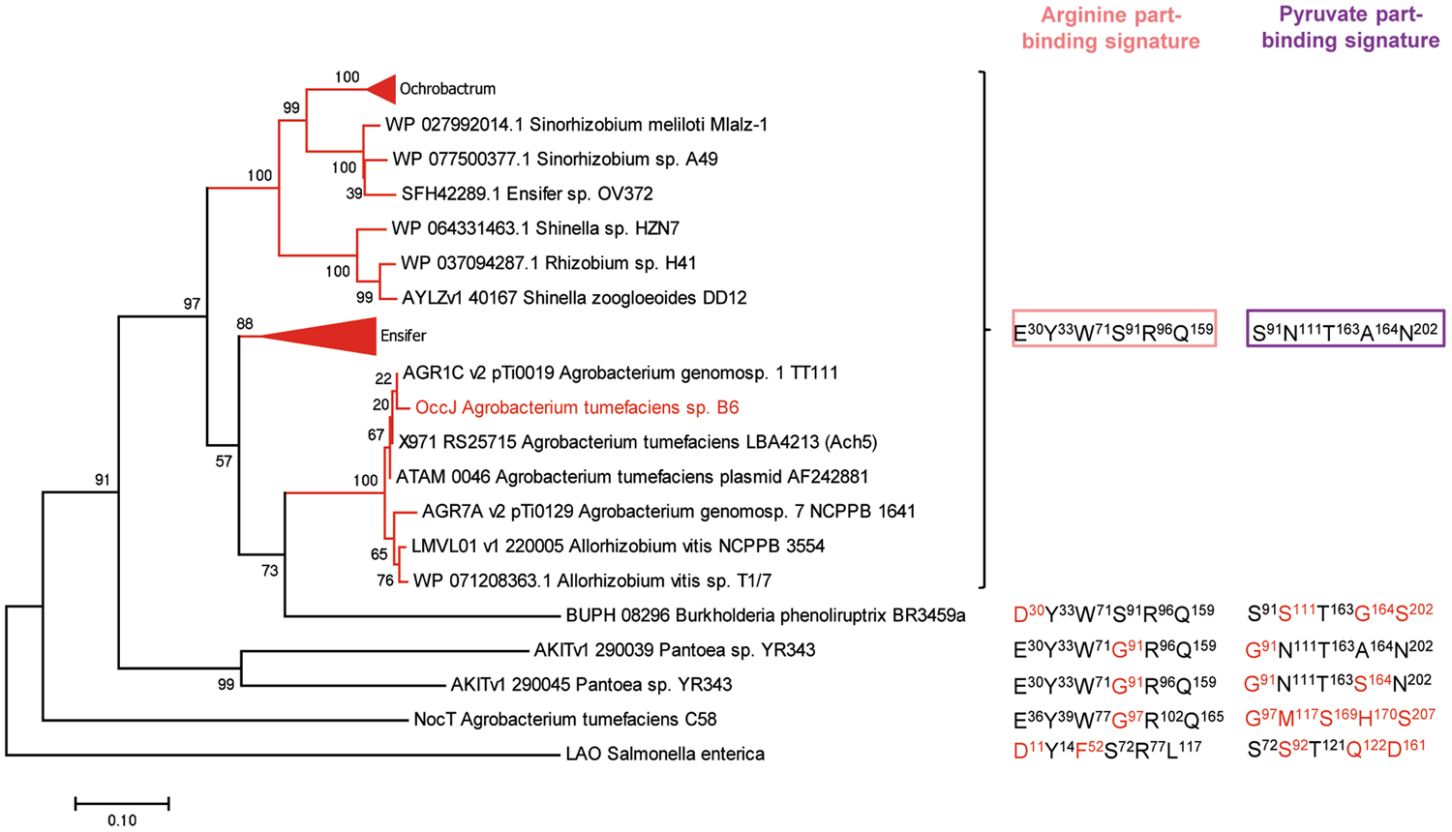
OccJ phylogeny and octopine binding signature. The displayed tree was rooted with *S*. *enterica* LAO sequence. For each protein or protein cluster, the residues which are identical to (black) and different from (red) those involved in the binding of the arginine part (pink box) and pyruvate part (magenta box) of octopine are indicated.

Using a threshold set at at least 40% identity, about 524 bacterial OccJ-homologous PBPs were recovered using blastP from NCBI bacterial sequence database and AgrobacterScope genome library (Genescope, France). All redundant sequences were removed, and the sequence of *S*. *enterica* LAO was added. The relation tree was built from 41 sequences (Fig. 5). Members of the OccJ subgroup sharing >69% sequence identity possess the octopine binding signature Glu30-Tyr33-Trp71-Ser91-Arg96-Gln159-Asn111-Thr163-Ala164-Asn202. They belong to five octopine-type *A*. *tumefaciens* (B6, AF242881, TT111, NCPPB 1641 and Ach5) and two *A*. *vitis* (T1/7 and NCPPB 3554) strains and to several strains belonging to soil and plant interacting genera *Sinorhizobium*, *Ensifer*, *Ochrobactrum* and *Shinella*. Remarkably one OccJ-like deduced protein from *Burkholderia phenoliruptrix* BR3459a might bind octopine with high affinity because its binding site differs by four similar residues: Asp30, Ser111, Gly164 and Ser202 are substituted for Glu30, Asn111, Ala164 and Asp202, respectively. Ser91, the critical residue, is conserved. Two paralogues from Enterobacteriales (*Pantoea*) would bind octopine but with an affinity in the micromolar range similar to the OccJ-S91G mutant due to the presence of G91 residue instead of Ser91. The octopine binding signature of OccJ is strongly degenerated in NocT and in LAO.

## Discussion

This work mainly focused on the study of the specificity of three related PBPs: OccJ and NocT, both encoded by the Ti-plasmid of octopine and nopaline-type *Agrobacterium* strains, respectively, and *S*. *enterica* LAO for Lysine-Arginine-Ornithine, belonging to the same PBP structural cluster.

At the molecular level, these PBPs share a similar overall ligand-binding site with several conserved residues around the amino acid part of the ligand but differ in their ligand selectivity and affinity. LAO is highly selective for the three amino acids with an affinity in the nanomalor range and does not bind any tested opine. OccJ displays high affinity for octopine (nanomolar range) and can bind the same amino acids than LAO but with an affinity in the micromolar range indicating that OccJ-mediated transport system has a strong preference for octopine. OccJ has no interaction with nopaline. In contrast, NocT does not bind amino acids and recognizes both octopine and nopaline with a very close affinity, in the micromolar range, underlying that NocT-mediated transport system has no preference between these opines. We previously defined four residues Gly97-Met117-His170-Ser207 as belonging to the nopaline-binding signature (Table 3,^14^). More precisely, these four residues interact with the ketoacid moiety of nopaline and octopine, making this nopaline-binding signature also an octopine-binding signature. Here, we established a structural basis for the high octopine specificity of the PBP OccJ and defined the high affinity octopine binding signature in which five residues form the binding signature of the octopine ketoacid (Table 3): Ser91-Asn111-Thr163-Ala164-Asn202. These residues are equivalent to Gly97-Met117-Ser169-His170-Ser207 in NocT belonging to the nopaline-octopine binding signature.

Site-directed mutagenesis of residues from this signature, combined with affinity measurements show that the residues at position 91/97 in OccJ/NocT have essential roles in each PBP. Ser91 is responsible for both the efficient binding of octopine at nanomolar range and the strong binding of amino acids. Replacing Gly97 with a serine lead to a selective octopine binding protein by restricting the opine volume. Indeed, the NocT mutant can no longer accommodate nopaline, which is bulkier than octopine. In LAO, none ketoacid binding signature is conserved in agreement with its ligand specificity, corresponding to Ser72-Ser92-Thr121-Gln122-Asp161. A Q122A mutation in LAO yields a protein able to bind octopine by disrupting the hydrogen bond between Asp161 and Gln122. Substitution of a single amino acid within the signature can drastically modify the protein function. In summary, we clearly demonstrate that to be a high affinity octopine binding protein, the presence of the serine within the octopine ketoacid signature is essential. In contrast, asparagine at position 202 is not a critical residue.

Our work eventually revealed the existence of two binding modes of octopine to PBPs, each associated with a signature as described above, the octopine binding signature of OccJ responsible for a highly efficient and selective import of octopine and the nopaline-octopine binding signature of NocT responsible for a less efficient and non-selective import. Both signatures allow the assimilation of other opines from the octopine family as well as that of the octopine derivatives. The structural data of NocT with octopinic acid, histopine and an octopine analogue confirm a unique binding mode with a unique conformation of the ketoacid moiety, which is different from that observed in OccJ. Though not demonstrated, it is tempting to assume that octopine family members and octopine analogues would bind OccJ with the same conformation and binding mode as octopine.

The above molecular data have physiological and ecological complements. In octopine-type *Agrobacterium*, we observed that the octopine content was higher in tumors induced by an *occJ* KO-mutant as compared to those induced by the WT. This shows that the octopine-type *Agrobacterium*, that expresses high selectivity and affinity (nanomolar range) octopine-PBP OccJ and the catabolic proteins encoded by genes located downstream *occJ*, efficiently exploits the octopine resource in plant tumor. In contrast, in nopaline-type *Agrobacterium*, we previously observed no difference in the nopaline content of plant tumors induced by the WT and a *nocT* KO-mutant^6^. This difference could be explained by the fact that the *nocT* KO-mutant, though unable to transport and grow on nopaline^6^, still expresses the nopaline catabolism genes. This trait differs in the *occJ* KO-mutant that is most likely unable to express the octopine catabolic genes. Alternatively, the two above observations could indicate that nopaline-type strains, which exhibit a lower affinity (micromolar range) to nopaline, might only partially exploit the nopaline resource in tumor. Several bacteria that do not belong to the *Agrobacterium* genus are able to import and degrade opines^21,22^. As a consequence, a high abundance of residual opine would facilitate their settlement and lead to a competition against agrobacteria in nopaline tumor as compared to octopine tumor.

The opine spectrum, that is exploited by *Agrobacterium* octopine-type and nopaline-type also differs. The selectivity of the PBPs NocT and OccJ (this work;^6,14^) and to a lesser extent that of opine-catabolic enzymes^23^ permit the assimilation of octopine only in octopine-type *Agrobacterium*, but that of nopaline and octopine in nopaline-type *Agrobacterium*. From an ecological point of view, the octopine-type exhibits the behaviour of a niche-exploitation specialist, while the nopaline-type that of a niche-exploitation generalist. The contrasting affinities of OccJ (nanomolar range) and NocT (micromolar range) for octopine could be a major ecological trait influencing the co-existence between opine niche-exploiting generalists and specialists. Furthermore, this would explain why nopaline-octopine generalists do not outcompete octopine specialists despite the advantage associated with broader nutritional niches^14^. In other word, the relatively high affinity of OccJ for octopine compared to NocT for octopine (~956 fold lower affinity) provides a tremendous advantage for *Agrobacterium* octopine-type when associated with octopine-producing tumors.

## Materials and Methods

### Synthesis of octopine, octopine derivatives and nopaline

Octopine and its structural relatives octopinic acid, lysopine and histopine were synthesised by condensations of L-arginine, L-ornithine, L-lysine and L-histidine respectively and L-2-bromopropionic acid^24^ while the analogue homo-octopine was synthesized from L-homoarginine and L-2-bromopropionic acid. Noroctopine, noroctopinic acid and homo-noroctopine analogues were synthetically obtained by the general method of Izumyia *et al*.^25^ involving condensation of L-arginine, L-ornithine and L-homoarginine respectively, with bromo-acetic acid in the presence of barium hydroxide as described by Petit and Tempé^26^. Nopaline was obtained by condensation between L-arginine and α-ketoglutarate in the presence of sodium cyanoborohydride as described by Tempé^24^.

### *A*. *tumefaciens* culture conditions

*A*. *tumefaciens* strain B6 and its derivatives were cultivated at 30 °C in Luria-Bertani modified medium (LBm with 5 g/L NaCl) or in *Agrobacterium* broth (AB) minimal medium (K_2_HPO_4_ 3 g/L; NaH_2_PO_4_ 1 g/L; MgSO_4_-7H_2_O 0.3 g/L; KCl 0.15 g/L; CaCl_2_ 0.01 g/L; FeS0_4_-7H_2_O 2.5 mg/L; pH 7) supplemented with ammonium chloride (NH_4_Cl, 1 g/L) and mannitol (2 g/L) except when alternative source of carbon and nitrogen is indicated. In growth assays, octopine was added as a sole carbon and nitrogen source at 1 g/L. The antibiotic gentamycin was added at 25 mg/L.

### Construction of *occJ* and *ocs* defective mutants in *A*. *tumefaciens* B6

The *A*. *tumefaciens* B6-ocs::Gm and B6-occJ::Gm defective mutants were constructed as described by Haudecoeur *et al*.^27^. Briefly, a gentamycin resistance cassette was inserted into the *ocs* and *occJ* genes, respectively, cloned into pGEM-T vector (Promega). The resulting plasmids were electroporated in *A*. *tumefaciens* strain B6 and marker exchange was selected using the gentamycin resistance trait. Both exchange mutants were verified by PCR. Though not verified, and based on previous data, the disruption of the *occJ* gene by the Gm cassette may exert a polar effect of the octopine catabolic genes located downstream in the sale transcription unit^13^.

### Plant infection

Tomato plants (F1 hybrid Dona, Vilmorin, France) were grown in greenhouse under long day conditions at controlled temperature (24–26 °C). Four-week old plants were wounded with a scalpel blade between the first and second stem nodes and inoculated with agrobacteria as described previously^28^. Plants were infected with a single genotype (*A*. *tumefaciens* B6 WT, B6-occJ::Gm and B6-ocs::Gm) or with a mixture of *A*. *tumefaciens* B6 WT and B6-occJ::Gm or *A*. *tumefaciens* B6 WT and B6-ocs::Gm at 20:80 and 15:75 inoculum ratio, respectively. Plant tumors were crushed into saline solution (NaCl 0.8%) to recover bacteria, which were spotted onto agar media supplemented with appropriate antibiotic to enumerate the colony forming units (CFU). The quantification of octopine was performed as previously described from macerates of whole tomato tumor^29^.

### Cloning of mature OccJ, NocT-G97S, OccJ-S91G, OccJ-N202D, LAO and LAO-Q122A

The mature OccJ expression plasmid was obtained by cloning *occJ* gene, without the twenty-five residues signal sequence, of *A*. *tumefaciens* B6 by PCR and adding a C-terminal hexahistidine tag into the plasmid pET-9aSN1 (a gift from S. Chéruel, I2BC, University Paris Sud, Orsay, France) between the *Nde*I and *Not*I sites using 5′-GGAATTCCATATGCAGGAGGAAAAGTCGATTACG-3′ as forward primer and 5′-TTTGCGGCCGCTCAATGGTGATGGTGATGGTGTTGGGGGGTGACA-3′ as reverse primer. The mutant NocT-G97S was generated using pET9a-SNI NocT^6^ as template, primers (5′-TATCTCCTCACGCCGAGTACGTTCTTG-3′ and 5′-CAAGAACGTACTCGGCGTGAGGAGATA-3′)with QuickChange II XL directed mutagenesis kit (Stratagen). The nucleotide sequences were confirmed by DNA-sequence analysis (GATC, France).

The mature LAO expression plasmid, LAO-Q122A, OccJ-S91G and OccJ-N202D were chemically synthesized using codon optimization for the expression in *E*. *coli* and inserted into pET-28a plasmid using *NotI* and *NdeI* restriction enzyme (Genscript, Piscataway, NJ).

### Protein production of mature OccJ, NocT-G97S, OccJ-S91G, OccJ-N202D, LAO and LAO-Q122A

*E*. *coli* BL21 competent cells transformed with each above recombinant plasmid were grown in 2TY medium at 37 °C until OD_600_ reached 0.6. Isopropyl β-D-1-thiogalactopyranoside (IPTG, 0.5 μΜ) was added to the culture for 4 h of expression at 28 °C. The cells were pelleted by centrifugation at 8,000 g for 20 min at 4 °C and resuspended in lysis buffer (50 mM Tris-HCl, 150 mM NaCl and 20 mM imidazole, pH 8) and disrupted by sonication. After centrifugation at 20,000 g for 30 minutes, the filtered supernatant was injected on a nickel affinity column (HiTrap 5 mL, GE Healthcare). After a washing step of 6% Buffer B (50 mM Tris-HCl pH 8, 150 mM NaCl and 300 mM imidazole), each protein was eluted with 100% Buffer B and injected on a gel filtration Superdex 200 26/60 (GE Healthcare) using 50 mΜ Tris-HCl pH 8 and 150 mM NaCl. The protein fractions were pooled, concentrated and stored at −80 °C.

To eliminate the bound arginine, LAO and LAO-Q122A were purified under denaturing/renaturing conditions as previously reported^20^. After cell lysis in denaturing buffer containing 50 mM Tris HCl pH 8, 10 mM imidazole and 7 M urea, the supernatant was loaded onto a nickel column equilibrated in the same buffer. Low-affinity binding contaminants were washed from the column with 50 mM Tris HCl pH 8, 20 mM imidazole, and 7 M urea. A buffer of 50 mM Tris HCl pH 8, 20 mM imidazole, and 500 mM NaCl was passed through the column to return the protein to renaturing conditions prior to its elution with 50 mM Tris HCl pH 8, 300 mM imidazole, and 500 mM NaCl. The eluted sample was further purified using a HiLoad 26/60 Superdex 200 prep grade (GE Healthcare) in 50 mM Tris-HCl pH 8 and 150 mM NaCl. The protein fractions are pooled, concentrated and stored at −80 °C.

### Crystallization and data collection

Crystallization conditions for unliganded OccJ (20 mg/mL) and OccJ-octopine (90 mg/mL protein and 6 mM octopine) were screened using Qiagen kits (Valencia, CA, USA) with a Cartesian nanodrop robot (Genomic solutions). The conditions are reported in Table 2. NocT and NocT-G97S were co-crystallized as previously described^6^. The crystals were manually reproduced in hanging drops experiments by mixing equal volumes of protein solution and precipitant solution. Crystals were transferred to a cryoprotectant solution (mother liquor supplemented with 25% PEG 400) and flash-frozen in liquid nitrogen. X-ray diffraction data sets were collected at 100 K on the Proxima 1 or 2 beamlines (SOLEIL synchrotron, Saint-Aubin, France).

### Structure determination and refinement

Data processing was performed using the XDS package^30^ (Table 2). All the structures were determined by molecular replacement with PHASER^31^. The crystal structures of both unliganded and liganded OccJ were solved using the coordinates of lobe 1 and lobe 2 of NocT monomer as separated search models (PDB code 4POW). The structures of the NocT and NocT-G97S mutant complexes were solved using the whole NocT-octopine structure (PDB code 5ITP). Refinement of each structure was performed with BUSTER-2.10^32^ with NCS restraints when the asymmetric unit contains more than one protein molecule. TLS group was assigned for each structure. Inspection of the density maps and manual rebuilding were performed using COOT^33^. The three dimensional models of octopine analogues were generated using the ProDRG webserver^34^. Refinement details of each structure are shown in Table 2. Molecular graphics images were generated using PyMOL (http://www.pymol.org).

### Fluorescence titration measurements

Binding of ligands to OccJ, NocT, LAO and protein mutants was monitored by autofluorescence by exciting the protein at a wavelength of 295 nm and monitoring the quenching of fluorescence emission of tryptophans at 335 nm. All experiments were performed at 20 °C in 96 wells plates (1/2 Area Plate-96F, Perkin Elmer) using Tecan Infinite M1000 reader (Tecan), in 50 mM Tris-HCl pH 8.0, 150 mM NaCl buffer with a fixed amount of proteins (1 µM) and increasing concentrations of ligand. No ligand exhibited an emission signal at 335 nm. The data were analysed using Origin^®^ 7 software and fitted to the equation f = ΔFluorescence_max_ * abs(x)/(*K*_*D*_ + abs(x)).

### Isothermal titration microcalorimetry measurements

Isothermal titration microcalorimetry experiments were performed with an ITC200 isothermal titration calorimeter from MicroCal (GE Healthcare). The experiments were carried out at 20 °C. Protein concentration in the microcalorimeter cell (0.2 mL) varied from 20 to 150 µM. Nineteen injections of 2 µL of ligand solution (amino acids, opines and octopine derivatives) at concentration ranging from 240 µM to 1.5 mM were performed at intervals of 180 s while stirring at 500 rpm. The experimental data were fitted to theoretical titration curves with software supplied by MicroCal (ORIGIN^®^). This software uses the relationship between the heat generated by each injection and ΔH (enthalpy change in kcal/mol), Ka (the association binding constant in M^−1^), n (the number of binding sites), total protein concentration and free and total ligand concentrations^35^.

### Phylogenetic analysis

Sequences were analyzed using blastP algorithm from NCBI (http://blast.ncbi.nlm.nih.gov/). Alignments of OccJ and related sequences were conducted using the ClustalW software. Relationship trees were constructed using the MEGA software, Version 5. Phylogeny was inferred using the neighbor-joining method. The bootstrap consensus tree inferred from 1,000 replicates was taken to represent the evolutionary history of the analyzed taxa. The evolutionary distances are in units of the number of amino acid substitutions per site.

### Accession Codes

Coordinates and structure factors have been deposited at the Protein Data Bank (PDB) under accession codes 5ORE for OccJ structure, 5ORG for OccJ-octopine structure, 5OT8 for NocT-G97S-octopine structure, 5OTA for NocT-octopinic acid structure, 5OTC for NocT-noroctopinic acid, 5OT9 for NocT-histopine structure and 5OVZ for the high resolution structure of NocT-nopaline complex.

## Electronic supplementary material

supp data
