# Supplementary material for: Structural basis for high specificity of octopine binding in the plant pathogen Agrobacterium tumefaciens
Source: Sci Rep. 2017 Dec 21;7:18033. doi: 10.1038/s41598-017-18243-8 (PMC5740067; doi:10.1038/s41598-017-18243-8)
Supplement: Supplementary file 1 — supp data [file 41598_2017_18243_MOESM1_ESM.pdf]

Structural basis for high specificity of octopine binding in the plant pathogen *Agrobacterium tumefaciens*

Armelle Vigouroux[1], Abbas El Sahili[1,$], Julien Lang[1&], Magali Aumont-Nicaise[1], Yves Dessaux[1], Denis Faure[1*] and Solange Moréra[1*]

Supplementary data: Figs. S1-S4 and Table S1

Fig. S1. ITC and fluorescence K_D measurements of OccJ, NocT, LAO and mutants towards octopine and amino acids. The top graphs show fluorescence monitoring of each protein upon titration with each ligand and fit (solid line) to a single binding model using Origin 7. Measures were done in triplicate. The lower graphs of each protein microcalorimetry experiments show heat differences upon injection of ligand (top panel) and integrated heats of injection with the best fit (solid line) to a single binding model using Microcal ORIGIN (low panel). Calculated parameters for each experiment are indicated.

Fig. S2. (a) Superposition of octopines bound to OccJ (in pink/magenta), to the wild-type NocT (in blue, PDB code 5ITP) and NocT-G97S mutant (in grey), (b) octopine in pink/magenta bound to the ligand binding site of NocT-G97S. Both positions of Ser97 in molecules A and B of the asymmetric unit are shown as S97 (A) and S97 (B). (b). Hydrogen bonds between NocT-G97S and octopine are shown as dashed lines in black (distances below 3.2 Å) and in magenta (distances between 3.2 and 3.4 Å). (c) superposition of octopine bound to NocT-G97S and nopaline bound to wild-type NocT (PDB code 4POX). Double arrows indicate distances.

Fig. S3. ITC and fluorescence K_D measurements of OccJ and NocT towards octopine family members and octopine analogues. The top graphs show fluorescence monitoring of each protein upon titration with each ligand and fit (solid line) to a single binding model using Origin 7. Measures were done in triplicate. The lower graphs of each protein microcalorimetry experiments show heat differences upon injection of ligand (top panel) and integrated heats of injection with the best fit (solid line) to a single binding model using Microcal ORIGIN (low panel). Calculated parameters for each experiment are indicated.

Fig. S4. Octopinic acid (a), noroctopinic acid (b) and histopine (c) bound to the binding site of NocT are shown in hot pink/magenta, hot pink/green, and red/magenta sticks, respectively, and in their annealing Fo-Fc omit map contoured at 3 σ. Hydrogen bonds between NocT and each ligand are shown as dashed lines in black (distances below 3.2 Å) and in magenta (distances between 3.2 and 3.4 Å). (d) superposition of the three ligands as well as octopine (PDB code 5ITP) in the binding site of NocT.

OccJ-octopine

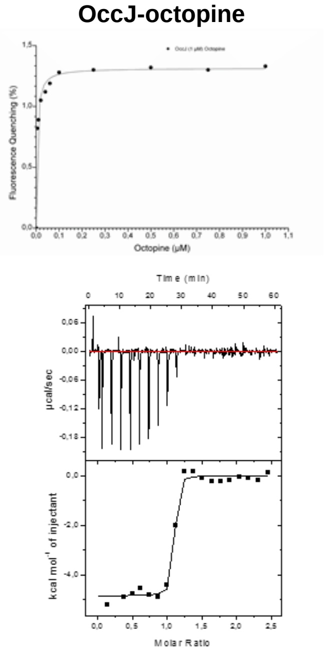

OccJ-L-arginine

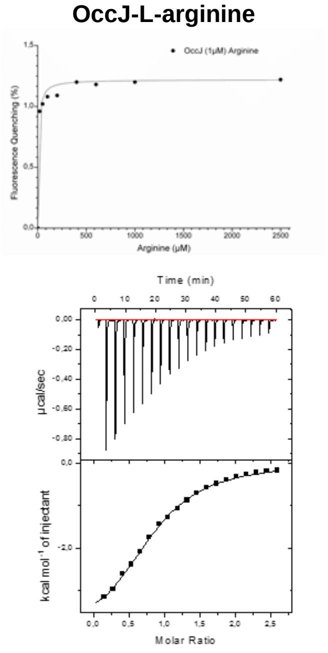

OccJ-L-lysine

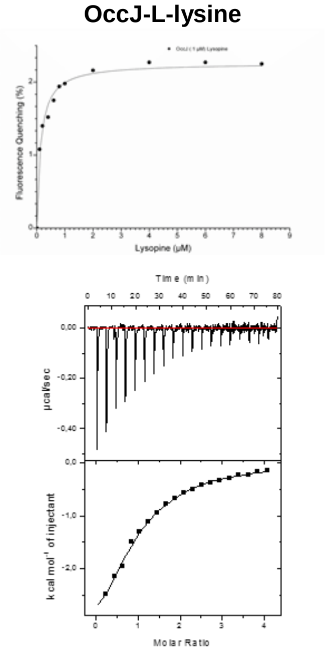

OccJ-ornithine

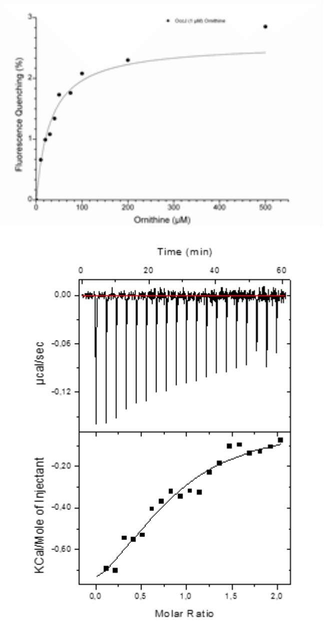

OccJ S91G-octopine

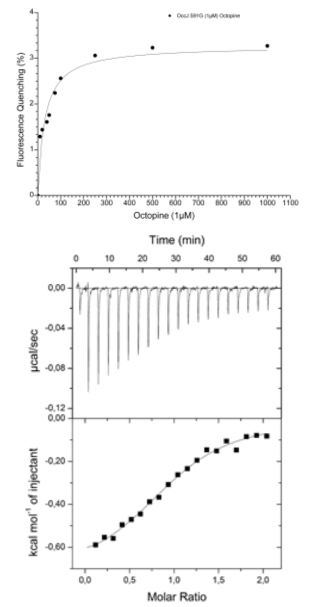

OccJ N202D-octopine

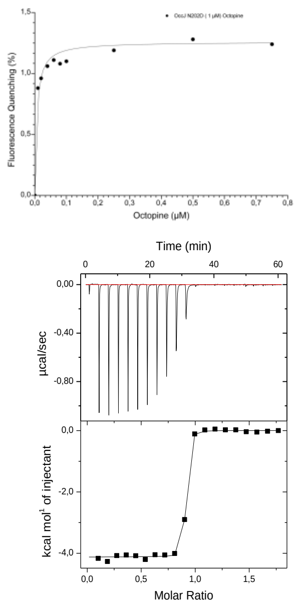

OccJ N202D-L-arginine

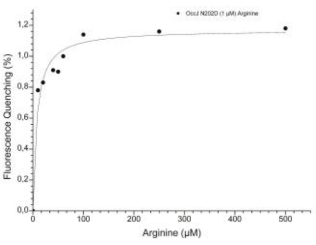

NocT G97S-octopine

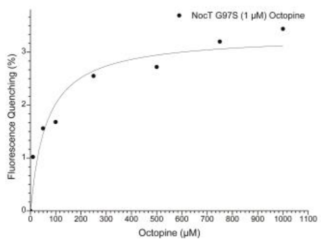

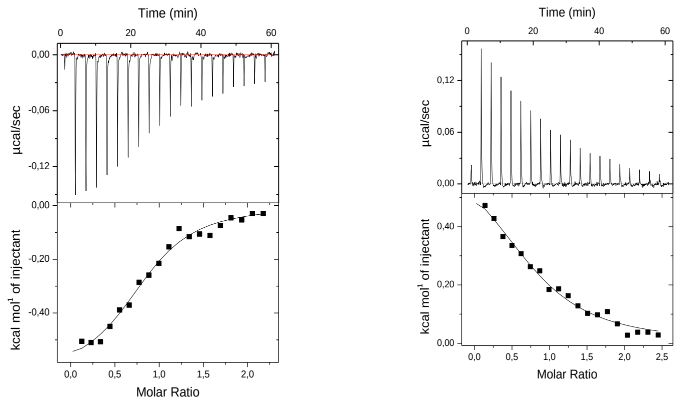

LAO WT-L-arginine **LAO Q122A-octopine** **LAO Q122A-L-arginine**

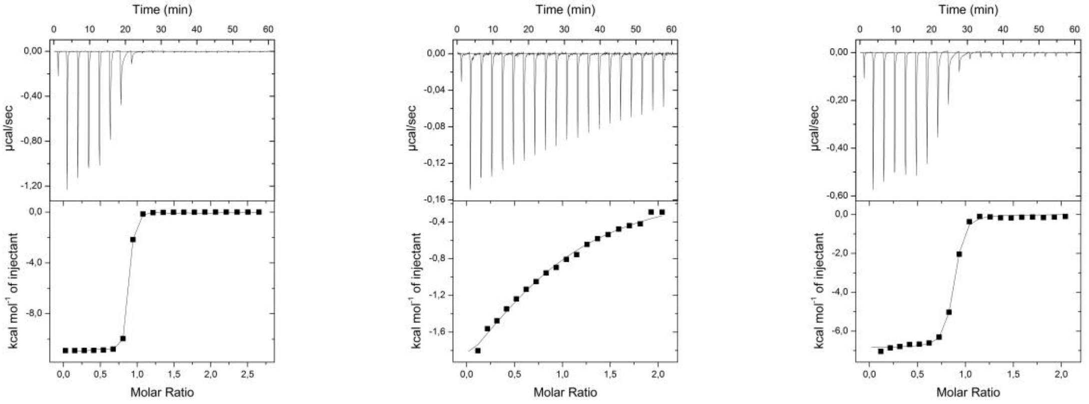

		ITC					
**Protein**	**Ligand**	**Kd (µM)**	**N**	**Enthalpy (ΔH) (cal/mol)**	**Entropy (ΔS) (cal/mol/ deg)**	**Entropic contribution (-TΔS) (cal/mol)**	**Free enthalpy (ΔG) (cal/mol)**
**OccJ WT**	octopine	0.009	1.04	-4837	20.4	-5980	-10817
	L-arginine	35.7 ± 2.1	0.84	-4208	5.99	-1755	-5964
	L-lysine	71 ± 6	0.87	-4920	2.20	-645	-5565
	ornithine	40.5 ± 15	0.80	-1102	16.3	-4778	-5880
**OccJ S91G**	octopine	20 ± 3	1	-726	19	-5569	-6295
**OccJ N202D**	octopine	0.016 ± 0.008	0.88	-4120	21.5	-6345	-10465
	L-arginine	20.4 ± 4	0.85	-632	19.3	-5696	-6328
**NocT G97S**	octopine	52 ± 10	0.83	687	21.9	-6420	-5733
**LAO WT**	L-arginine	0.024 ± 0.002	0.82	-10910	-2.4	703	-10207
**LAO Q122A**	octopine	34 ± 5	0.83	-3318	9.12	-2673	-5991
	L-arginine	0.12 ± 0.01	0.8	-6853	8.3	-2433	-9286

Figure S1

a

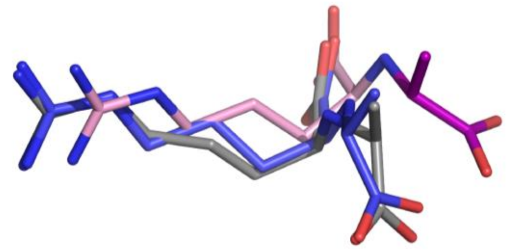

b

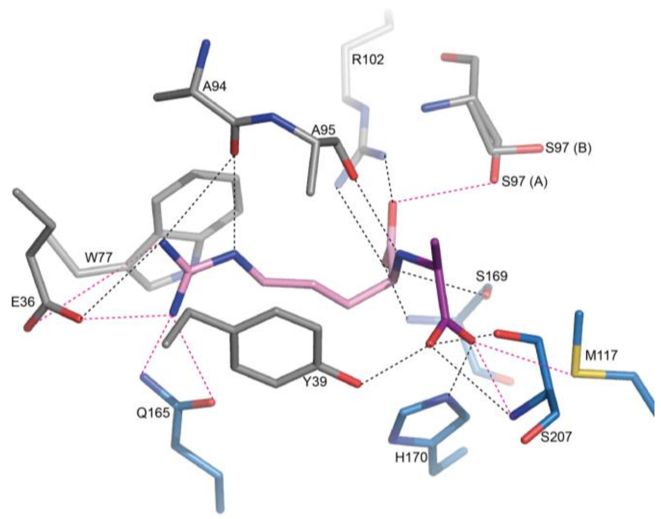

c

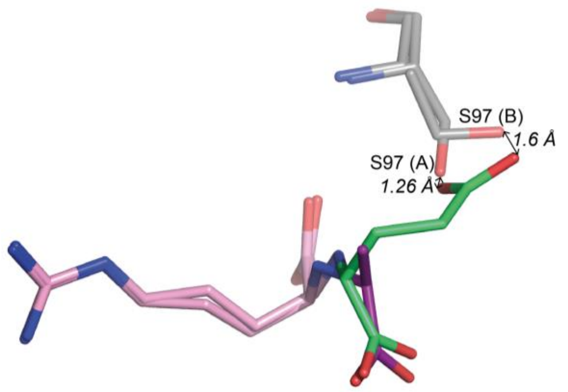

Figure S2

OccJ-octopinic acid

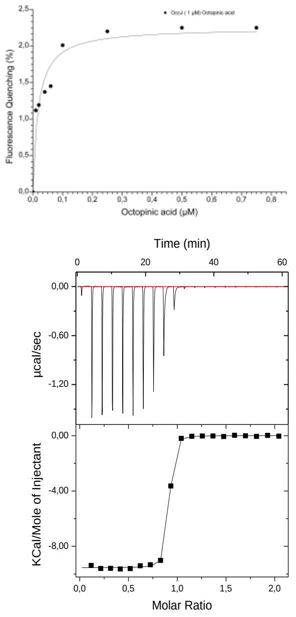

OccJ-lysopine

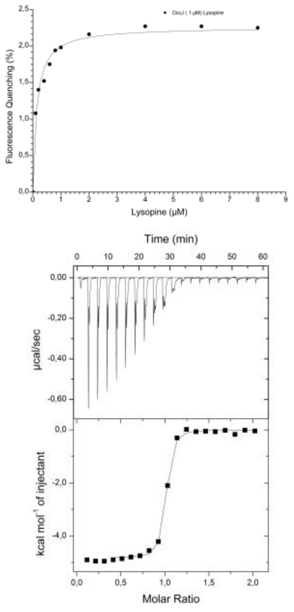

OccJ-histopine

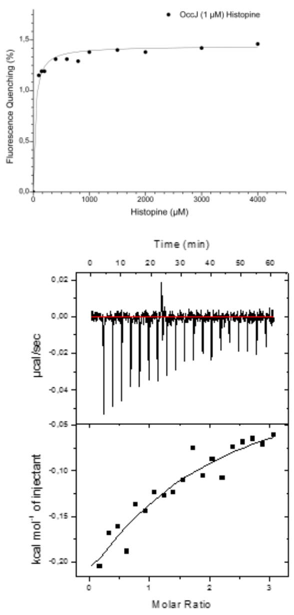

OccJ-homo-octopine

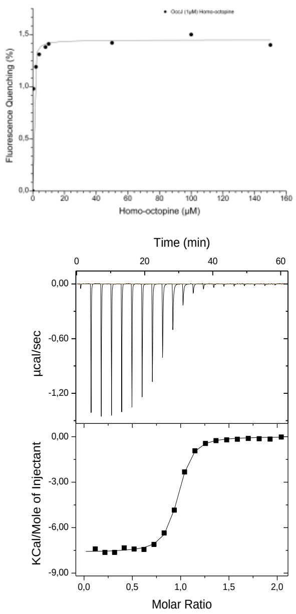

OccJ-noroctopine

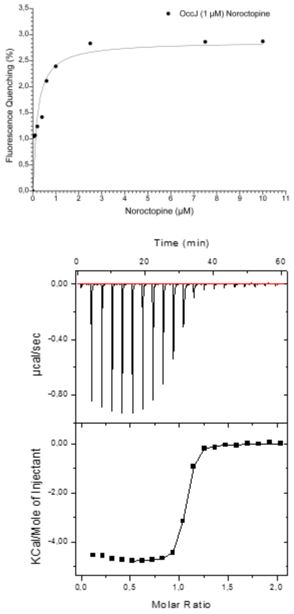

OccJ-noroctopinic acid

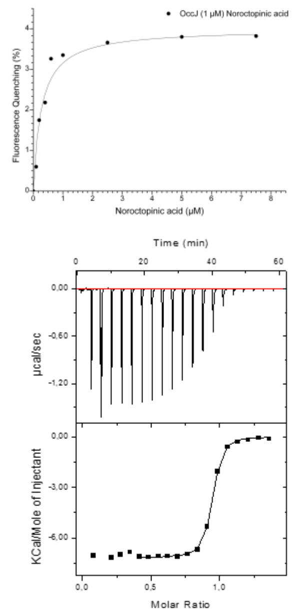

OccJ-homo-noroctopine

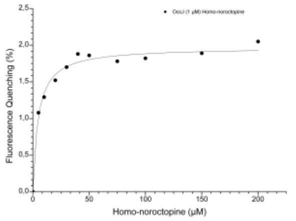

NocT-octopinic acid

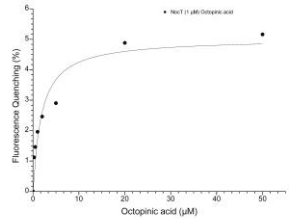

NocT-homo-octopine

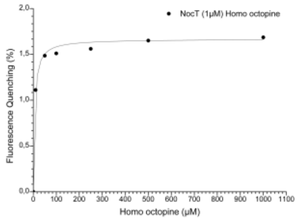

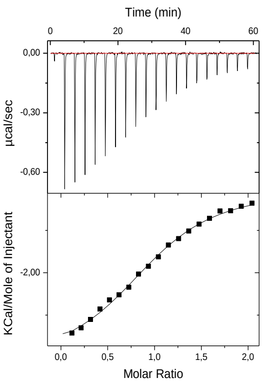

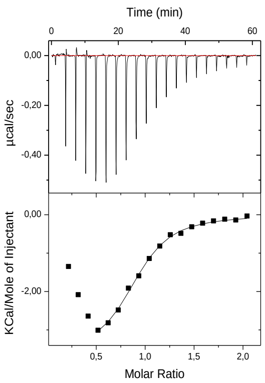

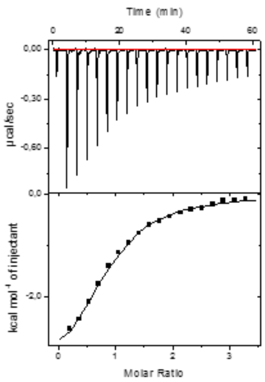

NocT-noroctopine **NocT-noroctopinic acid** **NocT-homo-noroctopine**

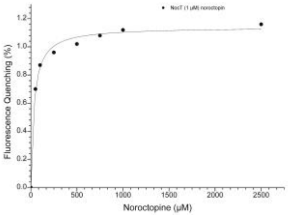

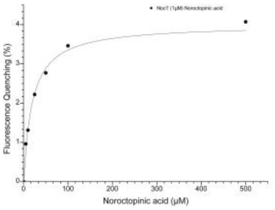

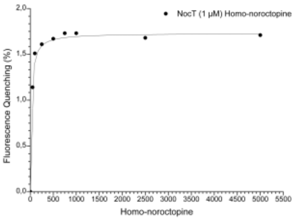

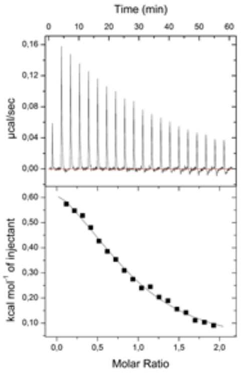

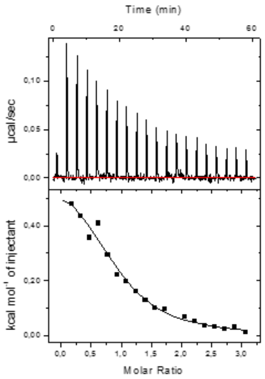

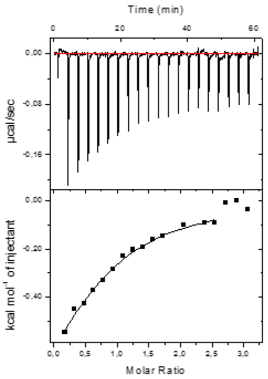

NocT-lysopine **NocT-histopine**

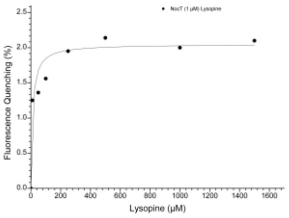

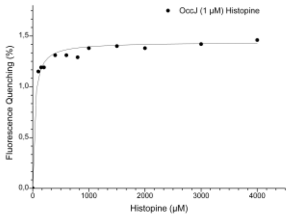

Figure S3

a

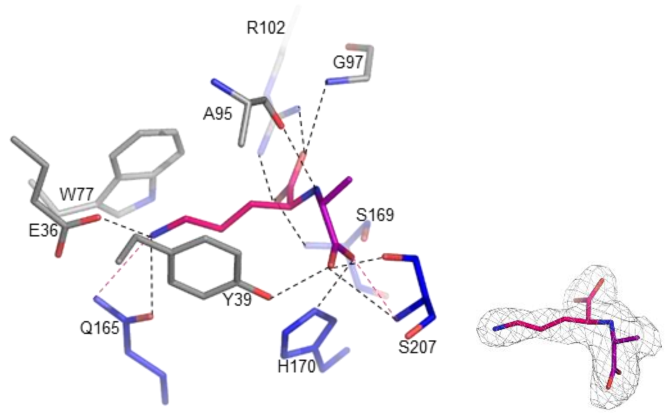

b

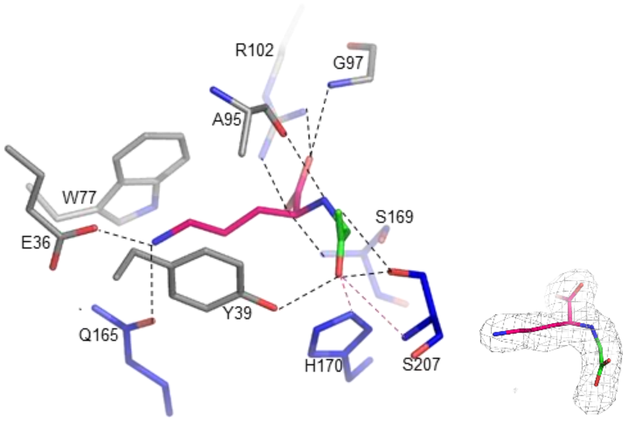

c

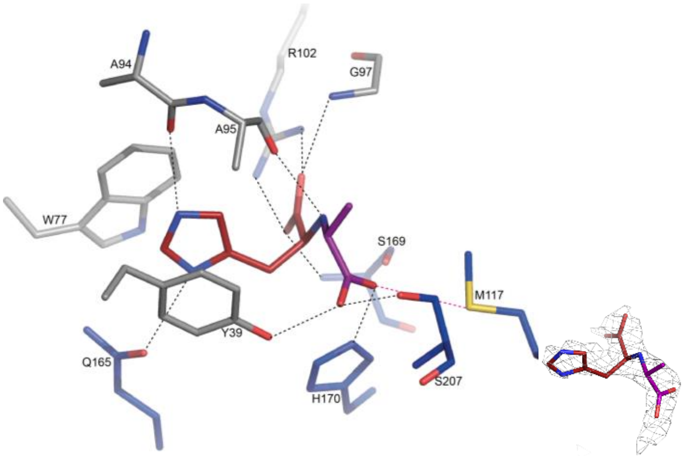

d

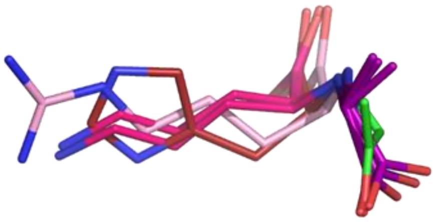

Figure S4

Table S1. Crystallographic data and refinement parameters of the high resolution NocT-nopaline structure.

	**NocT-nopaline**
PDB code	**5OVZ**
Precipitant	30% P4000/ 0.1 M Tris pH 8/ 0.1 M $LiSO_4$
Space group<br>Cell parameters (Å,°)	*P3₂*<br>*a* = 114.3<br>*b* = 114.3<br>*c* = 37.9
Resolution (Å)	50-1.75 (1.86-1.75)
No. of observed reflections	224742 (35655)
No. of unique reflections	55776 (8999)
R_{sym} (%) [a]	5.1 (83.7)
Completeness (%)	99.8 (99.5)
I/σ	14.2 (1.4)
$CC_{1/2}$	99.9 (57)
R_{cryst} (%) [b]	17.2
R_{free} (%) [c]	19
rms bond deviation (Å)	0.01
rms angle deviation (°)	1
Average B ($Å^2$)<br>protein<br>ligand<br>solvent	 38.5<br>30.1<br>49
